# Laparoscopic treatment of abdominal complications following ventriculoperitoneal shunt

**Published:** 2009-11-25

**Authors:** F Popa, VT Grigorean, G Onose, M Popescu, V Strambu, AM Sandu

**Affiliations:** * ‘ St. Pantelimon’ Clinical Emergency Hospital, Department of General Surgery, Bucharest Romania; ** ‘Bagdasar–Arseni’ Clinical Emergency Hospital, Department of General Surgery, BucharestRomania; *** ‘Bagdasar–Arseni’ Clinical Emergency Hospital, Department of Physical and Rehabilitation MedicineRomania; **** Pitesti District Hospital, Department of Neurosurgery, Pitesti Romania; ***** ‘Bagdasar–Arseni’ Clinical Emergency Hospital, Department Nerology, BucharestRomania

**Keywords:** abdominal complication, CSF ascites, CSF pseudocyst, laparoscopy, ventriculoperitoneal shunt

## Abstract

The aim of this study is the evaluation of laparoscopic treatment in abdominal complications following ventriculoperitoneal (VP) shunt.

**Methods**: We report a retrospective study including 17 patients with abdominal complications secondary to VP shunt for hydrocephalus, laparoscopically treated in our department, between 2000 and 2007.

**Results**: Patients' age ranged from 1 to 72 years old (mean age 25.8 years old). Male: female ratio was 1.4.

Abdominal complications encountered were: shunt disconnection with intraperitoneal distal catheter migration 47.05% (8/17), infections 23.52% (4/17) such as abscesses and peritonitis, pseudocysts 11.76% (2/17), CSF ascites 5.88% (1/17), inguinal hernia 5.88% (1/17), and shunt malfunction due to excessive length of intraperitoneal tube 5.88% (1/17).

Free–disease interval varies from 1 day to 21 years, depending on the type of complication, short in peritoneal irritation syndrome and abscesses (days) and long in ascites, pseudocysts(months– years).

Laparoscopic treatment was: extraction of the foreign body in shunt disconnection with intraperitoneal distal catheter migration, evacuation, debridement, lavage and drainage for pseudocysts, abscess and peritonitis, shortening of the tube in shunt malfunction due to excessive length of intraperitoneal tube a nd hernioraphy. One diagnostic laparoscopy was performed in a peritoneal irritation syndrome, which found only CSF ascites.

There were no conversions to open surgery. The overall mortality was of 5.88% and postoperative morbidity was of 11.76%. In 7 patients operated for abscesses, peritonitis, pseudocysts, and CSF ascites the shunting system was converted in to a ventriculocardiac shunt.

**Conclusions**: Abdominal complication following VP shunt can be successfully performed laparoscopically. Abdominal surgery required, in selected cases, the repositioning of the distal catheter, frequently as a ventriculocardiac shunt. There are abdominal complications with no indication of surgery, like peritoneal irritation syndrome and CSF ascites. Free– disease interval varies from days (peritoneal irritation syndrome, abscesses) to month–years (pseudocyst, ascites), according to type of complication.

## Introduction

Hydrocephalus is impairment in production, flow, or absorption of cerebrospinal fluid (CSF) that leads to an abnormal increase in CSF volume and, usually, pressure within the brain. Hydrocephalus is a health problem worldwide, with estimated prevalence of 1–1.5%. The incidence of congenital hydrocephalus is 0.9–1.8 new cases/1,000 births. [1] 

The term ‘hydrocephalus’ is derived from the Greek words ‘hydro’ meaning water, and ‘cephalus’ meaning head. Hippocrates described hydrocephalus for the first time, but it was not treated effectively until the middle of the 20^th^ century, when appropriate shunting techniques and materials developed.

The first ventriculoperitoneal (VP) shunt was done by Kausch in 1908, but the procedure did not become widely performed for more than 50 years, and since 1960, the technique has not changed much. In spite of all advances in neuroendoscopic surgery, the most common treatment for hydrocephalus remains VP shunt. VP shunt is widely preferred because of its well known advantages, such as: a potential infection of the shunting system has a lower systemic life–threatening risk compared to shunts into the venous system, in children, a large amount of tubing can be placed intraperitoneal, minimizing the need for elective lengthening with growth, the operation is safe, easy to perform and is not time consuming. 

Abdominal complications may occur, causing shunt dysfunction and acute hydrocephalus.

Distal VP shunt complications can be safely and effectively managed laparoscopically. This approach allows the distal catheter to be assessed and problems to be managed, thereby salvaging the existing shunt and avoiding the potential morbidity associated with additional VP shunt placement. [2] Via laparoscopic approach we can inspect the whole abdominal cavity and treat any associated pathology.

## Meth

We report a retrospective study including 17 patients with abdominal complications secondary to VP shunt for hydrocephalus, treated laparoscopically, between 2000 and 2007, in the Department of General Surgery from the Emergency Clinical Hospital Bagdasar–Arseni, Bucharest. Between 2000 and 2007 in the Department of Neurosurgery from the Emergency Clinical Hospital Bagdasar–Arseni, Bucharest 628 patients were treated for hydrocephalus by using VP shunt. Abdominal complications following VP shunt, occurred in 2.7%(17/628) cases. 

## Results

**Fig 1:**
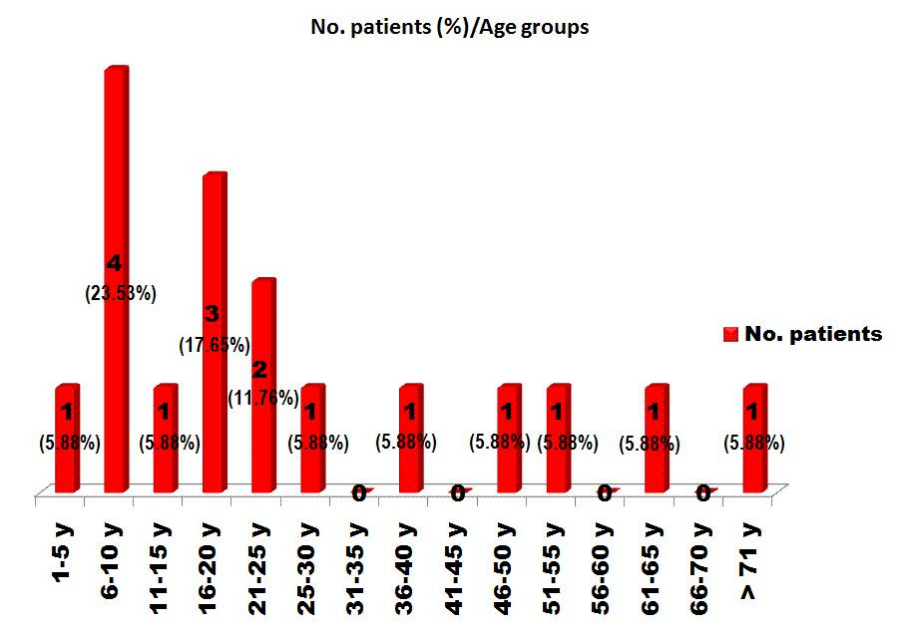
Patients' distribution according to age groups.

**Fig 2:**
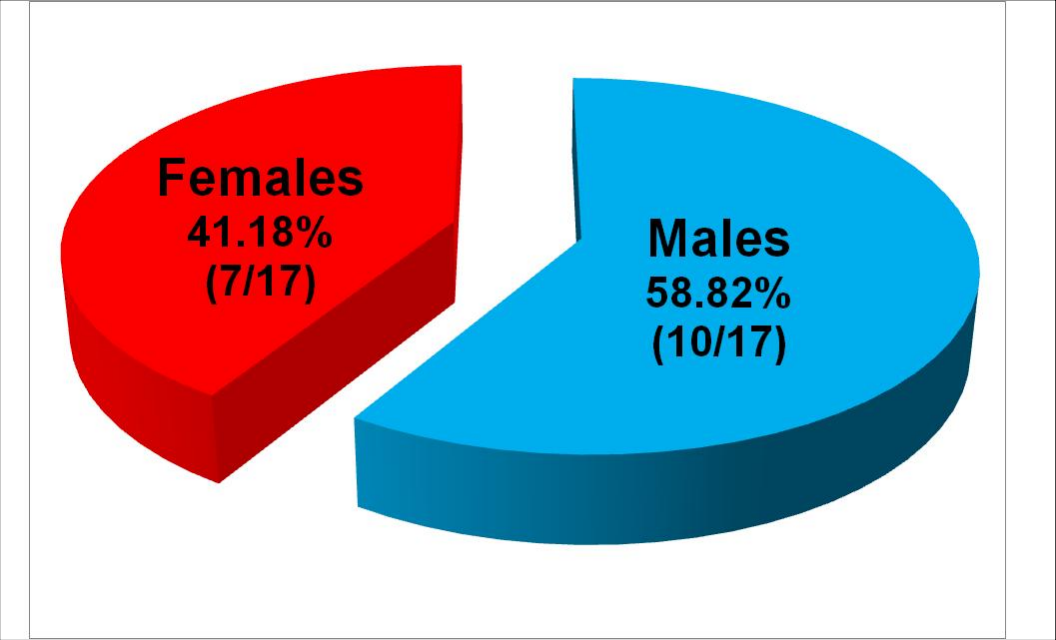
Patients' distribution according to sex.

*Patients' characteristics* Patients' age ranged from 1 to 72 years. Mean age was 25.8 years. The group consisted of 10 males and 7 females. Male: female ratio was 1.42.(Fig 1, Fig 2)

### Underlying neurosurgical disease

Five children, aged between 0 and 6 years old, had congenital hydrocephalus. Children with congenital hydrocephalus had other associated cerebroventricular malformations, like neural tube defects (myelomeningocele) in two cases, Dandy–Walker malformation in one case, Chiari I malformation in one case, and temporal bilateral and posterior fossa arachnoid cysts in one patient.

In young children secondary hydrocephalus was caused by posterior fossa pilocitic astrocytoma in three patients, medulloblastoma in one case, meningoencephalitis in one patient and craniopharyngioma in one case. In adults secondary hydrocephalus is caused by subarachnoid hemorrhage in two patients, one after ruptured aneurysm and one posttraumatic, and craniopharyngioma, ependymoma and posterior fossa hemangioblastoma, in the last three patients.

### Abdominal complications

The most frequently encountered abdominal complication following VP shunting in our group of patients was shunt disconnection with intraperitoneal distal catheter migration. It occurred in eight of the seventeen patients (47.05%). Distal catheter migrated into the pouch of Douglas in four cases, subhepatic in 2 cases, between small intestines in one patient and into the right paracolic space in one patient. Infection was found in four of the seventeen patients (23.52%). Three patients (17.64%) developed intraperitoneal abscess. According to abscesses location, one patient had abscess of lesser omentum (5.88%), anther one hepatic abscess (5.88%), and the last one peripancreatic abscess (5.88%). Two patients harboring abscess of lesser omentum and peripancreatic abscess had early infections. One patient with hepatic abscess developed late infection, within 2 years and 3 month following shunt insertion. Peritonitis was found in one case (5.88%) one day after the initial surgery. Pseudocysts were found in two patients (11.76%), and ascites with clear CSF was encountered in one patient (5.88%). Another patient developed an inguinal hernia without hydrocele (5.88%). Shunt malfunction was caused in one case (5.88%) by excessive length of the intraperitoneal tube.(Table 1)

**Table 1:** Abdominal complications following VP shunt

Diagnostic	No. patients	%value
Shunt disconnection with intraperitoneal distal catheter migration	8	47.05%
Abscess	3	17.64%
abscess of lesser omentum	1	5.88%
hepatic abscess	1	5.88%
peripancreatic abscess	1	5.88%
Peritonitis	1	5.88%
Pseudocyst	2	11.76%
CSF ascites	1	5.88%
Inguinal hernia	1	5.88%
Shunt malfunction due to excessive length of intraperitoneal tube	1	5.88%

**Fig 3:**
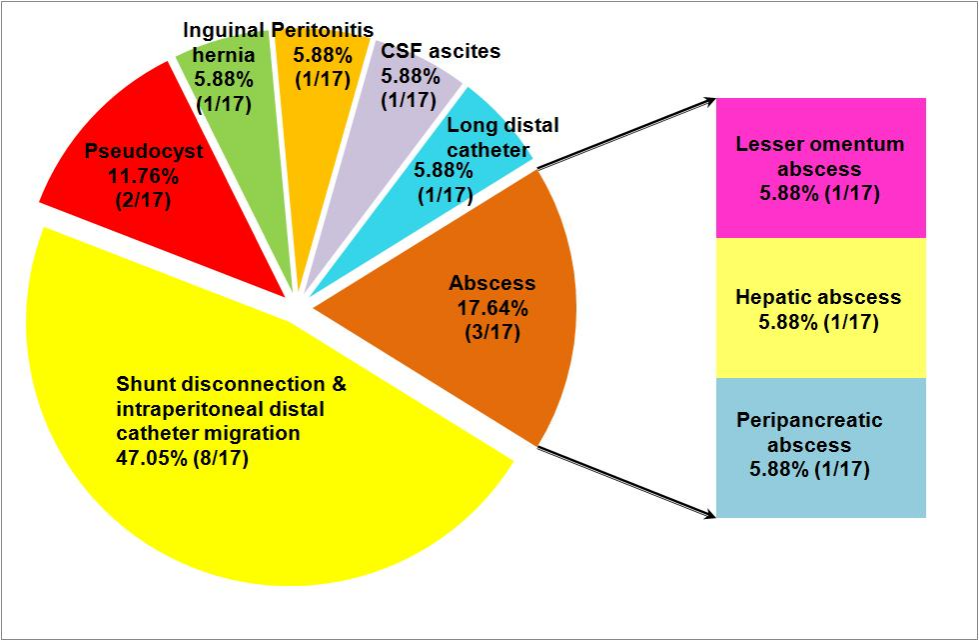
Abdominal complication following VP shunt

**Fig 4:**
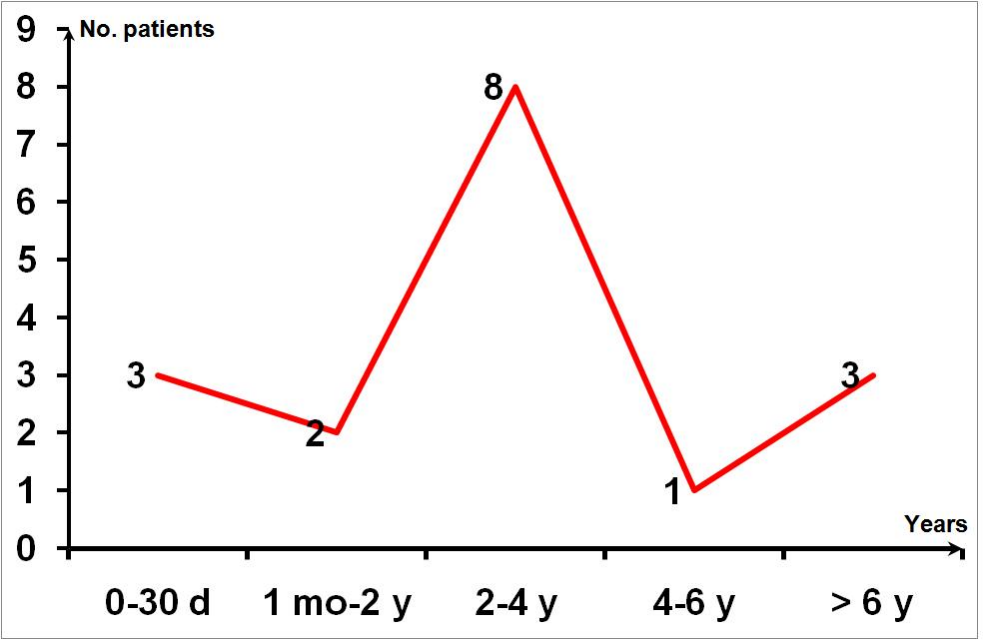
Free–disease interval

Free–disease interval, the period of time from shunt insertion to abdominal complication occurence, varies from 1 day to 21 years, depending on the type of complication. It is short in peritoneal irritation syndrome and abscesses (days) and long in ascites, pseudocysts (months–years). (Fig 3, Fig 4)

### Clinical findings,Neurological findings

Almost all patients presented symptoms suggesting an elevated ICP. Headache was encountered in sixteen patients (94.11%), sixteen patients had also vomiting (94.11%) and 9 cases presented loss of consciousness (52.94%). Four patients (23.52%) presented seizures, in one case (5.88%) were new onset seizures, and in three cases (17.64%) with prior epilepsy seizures increased in frequency and were uncontrolled by usual antiepileptic therapy. Cranial nerve palsy, such as abducens palsy and upward gaze palsy occurred in six cases (35.29%). Four patients presented recent progression of prior neurological deficits (23.52%).

### Abdominal symptoms

Diffuse abdominal pain was the most common abdominal symptom found in eleven cases (64.70%). One patient had localized abdominal pain in the groin area (5.88%). Ten patients presented abdominal tenderness (58.82%), four abdominal wall rigidity (23.52%), and one abdominal wall distension (5.88%). Paralytic ileus was found in two patients (11.76%) with peritonitis and CSF ascites. An abdominal mass was discovered in two cases (11.76%) on palpatory examination, both cases confirmed afterward to have pseudocysts and a bulge in the groin in one (5.88%) who was diagnosed with inguinal hernia.

Other clinical findings were dyspnea in two patients (11.76%) and fever in three cases (17.64%). Dyspnea was present in the two patients with paralytic ileus. Fever was a common finding among patient with early abscesses and peritonitis, all three of them presenting fever.(Table 2)

**Table 2:** Clinical findings

Diagnostic	No. patients	%value
**NEUROLOGICAL FINDINGS**		
Elevated ICP symptoms	17	100%
Headache	16	94.11%
Vomiting	16	94.11%
Loss of consciousness	9	52.94%
Seizures	4	23.52%
Cranial nerve palsy	6	35.29%
Progression prior of neurological deficits	4	23.52%
**ABDOMINAL SYMPTOMS**		
Abdominal pain	11	64.70%
Pain in the groin area	1	5.88%
Abdominal tenderness	1	5.88%
Abdominal wall rigidity	1	5.88%
Abdominal wall distension	1	5.88%
Abdominal mass	2	11.76%
Bulge in the groin	1	5.88%
Ileus	2	11.76%
**DYSPNEA**	2	11.76%
**FEVER**	3	17.64%

### Paraclinical findings

All patients underwent cerebral CT–scan, fundoscopy, abdominal ultrasonography, abdominal CT–scan and plain X– rays of cranium, thorax and abdomen. CSF analysis and CSF microbiological cultures were done in the seven patients with abscesses, peritonitis, pseudocysts and ascites. Fundoscopy performed in all seventeen patients, showed signs of incracranial hypertension, like papilledema.

**Fig 5:**
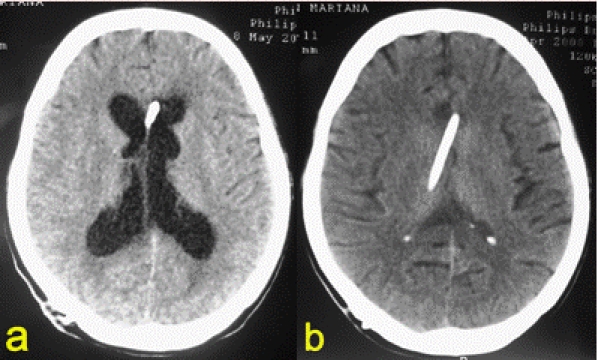
Cerebral CT. a. preoperative; b. postoperative

Cerebral CT–scan shown in all cases sings of active hydrocephalus, such as ventricles enlargement and transependymal absorbtion. Lateral ventricles were enlarged, with visible temporal horns and ballooning of the frontal horns. The third ventricle was also enlarged and periventricular hypodensity, a sign of transependymal absorption of CSF were also present. Evan's ratio was > 30% in all cases. The proximal end of the ventricular catheter was located within the lateral ventricle and no signs of proximal obstruction of the shunt can be seen on cerebral CT–scan. Postoperative CT–scan showed ventricular shrinking.(Fig 5)

Abdominal ultrasonography and CT–scan helped us establish the diagnosis rapidly and noninvasively.

Abdominal ultrasonography revealed intrabdominal transonic cystic mass in two patients, suggesting gross amount of encysted fluid within, and ascites in one case. Intraperitoneal fluid, a sign of good shunt functionality, was not noted, except for the one case with ascites.

**Fig 6:**
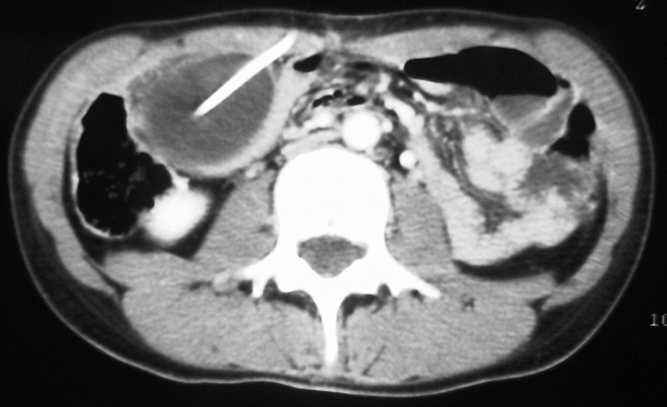
Abdominal CT. CSF pseudocyst with distal end of the peritoneal catheter within it.

**Fig 7:**
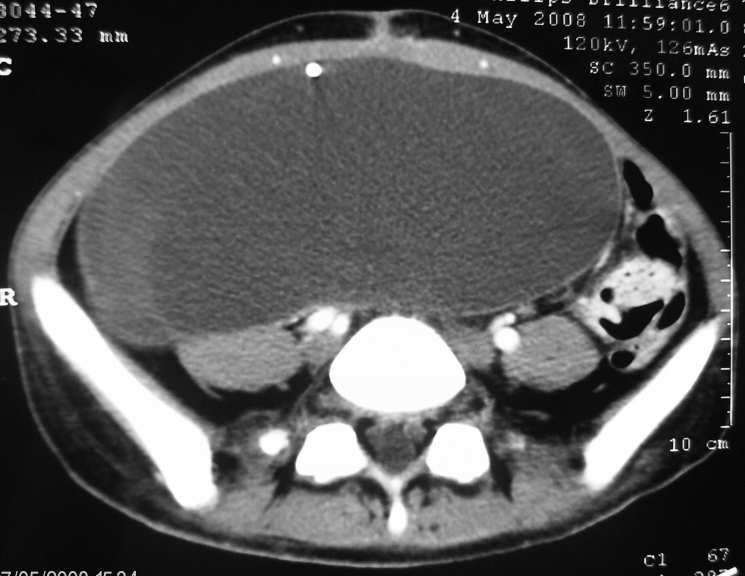
Abdominal CT. Gyant CSF pseudocyst

CT–scan showed intrabdominal foreign body (free distal catheter) in eight cases, hypodense intrabdominal mass containing tip of shunt catheter within it in two cases, and intraperitoneal ascites fluid in one patient.(Fig 6, Fig 7) 

Plain X–rays of cranium, thorax and abdomen showed shunt devices disconnection and migration of the distal catheter in eight cases, and shunt integrity in other nine cases.

CSF analysis showed high cellularity/mm [3]  in cases with infection and a progressive decease with antibiotherapy.

Microbiological cultures isolated staphylococci in all three cases with early infections, and enterococci in one late infection. 

### Treatment

**Fig 8:**
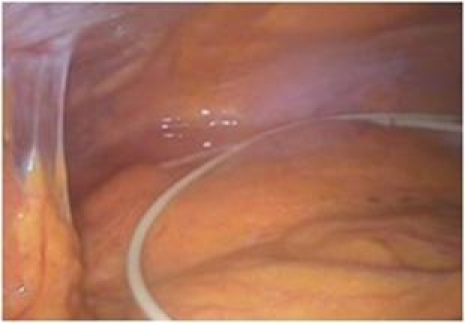
Shunt disconnection with distal catheter migration. Free intraperitoneal distal catheter.

In all eight patients with shunt disconnection and intraperitoneal distal catheter migration extraction of the foreign body was performed laparoscopically. A new distal catheter was inserted. (Fig 8)

In the three patients with abscesses and peritonitis we performed evacuation of the abscess, debridement, lavage, drainage. An external ventricular shunt was performed, until three consecutive sterile CSF culture with a cellularity beneath 5 cells/mm [3], allowed us to convert the shunt into a ventriculocardiac one. Patients received aggressive systemic antibiotherapy. Time need for the CSF to become sterile and with poor cellularity was 10, 13, 15, 26 days, respectively.

**Fig 9:**
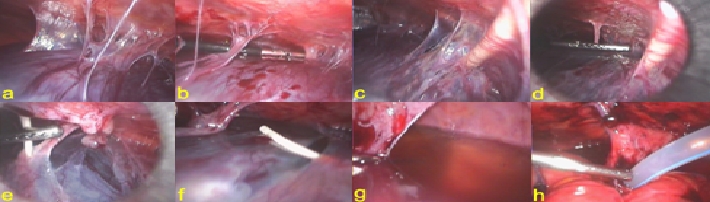
CSF pseudocyst. a. general view of the pseudocyst; b. adhesiolysis; c. distal catheter entering the pseudocyst; d. e. adhesiolysis of the distal catheter; f. distal catheter freed from the pseudocyst; g. partially evacuated pseudocyst; h. drainage of the pouch of Douglas.

In the two patients with pseudocysts evacuation, debridement, lavage and drainage were done, followed by external ventricular drainage and later, within 3 days, a ventriculocardiac shunt. (Fig 9) 

One case with peritoneal irritation syndrome we performed a diagnostic laparoscopy who found only CSF ascites. Further treatment was ventriculocardiac shunt.

Hernioraphy was performed in one patient with inguinal hernia. Taking into consideration the fact that the shunt was fully functional it was left in place.

The patient found to have shunt malfunction due to excessive length of intraperitoneal tube, required shortening of the tube.  

### Outcome

We had no conversions to open surgery.

Morbidity rate in our group of patients was 11.76%, two of the seventeen patients developing complications. Two patients develop fever and wire granuloma, respectively.

Mortality rate was 5.88%. The patient died not because of abdominal cause, by of the underlying disease, which was tumor recurrence.

## Disscusion

### Patients' characteristics

In our study patients' age varied between 1 year and 72 years. Mean age of 25.8 years show the preference of this disease to affect young people. Taking into consideration the free disease interval, between shunt insertion and complication occurrence, we find that the most affected age groups by congenital hydrocephalus is 0–6 years. Older patients had secondary hydrocephalus.

### Underlying neurosurgical disease

Congenital hydrocephalus was found in children, aged between 0 and 6 years old, and all of them had other associated cerebroventricular malformations, like neural tube defects (myelomeningocele), Dandy–Walker malformation, Chiari 1 malformation, and temporal bilateral and posterior fossa arachnoid cysts.

Causes that lead to secondary hydrocephalus differ according to age. In young children secondary hydrocephalus was caused mostly by specific tumoral pathology to this age, such as pilocitic astrocytoma and medulloblastoma. In adults, secondary hydrocephalus was caused by subarachnoid hemorrhage, after ruptured aneurysm and posttraumatic, craniopharyngioma, ependymoma and hemangioblastoma.

### Abdominal complications and their treatment

In our study group the most frequently abdominal complication following VP shunting was shunt disconnection with intraperitoneal distal catheter migration, occurring in 47.05% cases. Migration, the most frequently encountered shunt–related complication, is due to high mobility of the distal catheter and anatomical features of the abdominal cavity. Catheter migration may occurs into the abdominal cavity [3, 4], abdominal wall [3, 4], mouth [5], small bowel [3, 6], colon [3], stomach [3, 6], anus [5, 6], gallbladder [7], liver [6], urinary bladder [3, 6], urethra [5], scrot [3, 5, 8], vagina [3, 5, 6, 9], ombilicus [3, 5], pleural cavity [10– 12], mediastinum [3], heart [13]  and big vessels [3, 13]. Persistent patency of peritoneal folds, such as unobliterated processus vaginalis or median umbilical ligament, together with positive abdominal pressure can cause catheter migration into the scrotum [3, 5, 8]  or ombilicus [14]. Respiratory movements cause migration into the thorax, by creating intermittent negative abdominal pressure. [4, 10, 15]  Risk is higher if the patients harbor congenital diaphragmatic hiatuses, foramen of Morgagni or foramen of Boschdalek, which might allow a prolapse of the peritoneal catheter into the thoracic cavity. [11, 12]  Positive abdominal pressure, negative intraventricular pressure, and flexion– pextension movements of the neck facilitate proximal migration of the catheter. [3, 16]  Proximal migration occurs less frequently, in 0.1-0.4% cases, into the lateral ventricles, into the subdural, subarachnoid or subgaleal space or intraparenchimatal. [3, 6, 17]

With the advent of one-piece shunts the incidence of this type of complication deceased dramatically. [3]  However, in our series all patients had only multi–pieces shunts. Multi–pieces shunt allows separate replacement of either proximal or distal catheter, during shunt revision. By using one–piece shunt we can decrease the rate of abdominal complication with 47.05%.

Multi–pieces shunt can disconnect at any attachment site. Shunt disconnection in our group was in seven cases at the interface of the silicone tube and metallic connective piece, and in one case rupture of the silicon tube, at distant site to the metallic piece. The weakest points are the interface between silicone and metallic components and ligatures, which favor tearing of the plastic material. [3]

Usually, in shunt disconnection without migration of either proximal or distal catheter, reconnection of the shunt devices is the treatment of choice, if there are no signs of infection. [4] No patients in our group fit the criteria, all of them being found to have distal catheter migration. Distal catheter migrated into the pouch of Douglas in half of the patients, subhepatic, between small intestines and into the right paracolic space in the other half. The pouch of Douglas is known to be prone for intraperitoneal foreign bodies' migration, because it's declining position. The same tendency is found in our group of patients, half of them having distal catheter migration into the pouch of Douglas. 

Although, unlike ventriculoatrial shunts, when distal catheter is mandatory to be removed because of the risk of developing fatal cardiac arrhythmias or migration into the pulmonary artery, in VP shunts distal catheter can be left in place if there is no sign of infection. [3]  However, we do not recommend it, because of the high risk of further migration, visceral perforation, bowel obstruction or infection, that can require a new operation. [4, 18– 20]  We strongly recommend extraction of the distal catheter. When performing a standard shunt revision, because of the distant migration of the distal catheter, it is impossible to remove the intraperitoneal tube. Via laparoscopic approach we can inspect the whole abdominal cavity and easily find and extract the distal catheter. 

Visceral perforations are rare, but lethal complication, and can occur up to 10 years after initial surgery. [21]  The mortality of visceral perforation in shunted patients reaches 15%. [3]  Most likely visceral perforation occur into bowel [3, 6, 22– 24], gallbladder [7], stomach [21, 24– 26], urinary bladder [3, 6], scrotum [3, 5, 8, 14], vagina [3, 5, 6, 9]  etc. Children with various central nervous system (CNS) malformations that associate hydrocephalus, such as neural tube defects (myelomeningocele), are prone to visceral perforations because of the high friability of visceral wall and low visceral mobility. [4] Pathophysiology of visceral perforation is not clear yet. Probably it is the result of local inflammatory process as a response to a foreign body, favoring adherence to viscera. Visceral wall is thinned and finally perforated by repeated movements, resulting in localized or generalized peritonitis. [27] Usage of new modern soft silicone catheters decreased the rate of visceral perforation.

Bowel obstruction is another unfortunate complication with abandoned intraperitoneal distal catheter. Incriminating factors favoring ileus occurrence are bowel volvulus around intraperitoneal catheter and adherences between catheter and intestines secondary to the local inflammatory process as a response to a foreign body. [20] Ileus secondary to abandoned catheter should not be mistaken with other pathology, specific to infants and toddlers, causing bowel obstruction, like invagination of one portion of the intestine into another and volvulus.

Infections occurred in our series of patients in 23.52% cases, 17.64% developed intraperitoneal abscess, abscess of lesser omentum, hepatic abscess, and peripancreatic abscess and 5.88% peritonitis. 

Infections are among the main causes of shunt dysfunction [28], occurring in 0.17–30% of shunted patients. [6] They usually became symptomatic rapidly after shunt insertion, 70% of them being diagnosed within the first month. By 9 month 90% of shunt infection became clinical. [29]

Predisposal factors are prematurity, small age (< 1 year old), low birth weight, history of shunt dysfunction, long operating time, fluid accumulation along shunt tract, CSF leaks, history of intraventriclar hemorrhage or CNS infections, myelomeningocele, hyperproteinnorrachia, immunosuppression, previous abdominal surgery, and prolonged hospital stay. [3, 28, 30]

Baird et al. suggested differences between early and late infections, occurring after 9 month from shunt insertion. Early infections usually are due to contamination during surgery, while late infections are seeded from an abdominal site. [31, 32]  He found in early infections germ like *Staphylococcus epidermidis* (52.8–88.9%) and *Staphylococcus aureus* (12–40%). In late infection he found *Propionibacterium acnes, Enterococcus and Streptococcus faecalis*, but no staphylococci. [3] In our study group, we had three patients (17.64%) with early infection, abscess of lesser omentum, peripancreatic abscess and peritonitis, and one patient with late infection, hepatic abscess occurring 2 years and 3 month following shunt insertion. Microbiological cultures isolated staphylococci in all cases with early infections, and enterococci in late infection.

Early infections can be prevented by the following: shunt procedure must be the first operation on the schedule in that operating room, limited access into the operating room, minimizing operation time, small skin incisions, valve positioning at distance to skin incision, prophylactic antibiotherapy, immunoglobulin prophylaxis, antibiotic–impregnated catheters, multiple layers closure of the wound. Haines et al. using prophylactic antibiotherapy deceased infection rate with 50% [33], but Schurtleff et al. found no improvement. [19]  Usage of clindamycin and rifampicin impregnated catheters offered an effective protection against staphylococci for 42–56 days and decreased infection rate at 6 month from 12% to 1.4% [28, 34]  Ersahin et al. using immunoglobulin prophylaxis (sandoglobulin 1g/kg, 12 hours prior operation) had an infection rate of 0% immediately postoperative and 6.6% at 6 month. [35] Regarding our late infection, intrahepatic migration of distal catheter causing liver abscess is considered to be a very rare complication. The pathogenesis of liver abscess usually involves direct erosion of the liver by the distal catheter and translocation of gut flora along the tube. [36– 38] In our patient, the most likely pathogenic mechanism was direct erosion of liver by VP shunt tube. 

There were two tends in treatment of shunt infections. Walters et al. recommend antibiothepary alone in cases with functional shunt system and surgery in cases with shunt dysfunctions. [39] Although this might avoid a surgery, most authors believe that antibiotherapy alone cannot treat infection and recommend external ventricular drainage, antibiotherapy and shunt reintegration. Our opinion is that the first step should be external ventricular drainage and aggressive systemic antibiotherapy. Although there are authors that recommend intraventricular antibiotherapy [40], we consider systemic antibiotherapy to be sufficient. CSF samples taken daily were examined for cellularity. Three consecutive sterile CSF culture with cell number/mm^3^ beneath 5/mm [3], allowed us to convert the shunt into a ventriculocardiac one. Ventriculocardiac shunts were preferred to inserting the distal catheter into a former septic peritoneal cavity. We prefer this variant because septic process led to adherences, bride and fibrous reshuffling between abdominal viscera and predispose to distal shunt malfunctions and pseudocyst formation in the future. Special care should be taken with patients harboring ventriculocardiac shunts, and they should receive prophylactic treatment for sepsis and glomerulonephritis. 

Laparoscopy allowed us to treat associated lesion, in our case liver abscess. Plus, in all cases with secondary infections visceral lesions can be managed successfully via laparoscopic approach.

CSF pseudocysts were found in our series in 11.76% cases. They are rare complications of VP shuns, occurring in 1– 4.5% cases. [41– 44] Laparoscopic treatment of a CSF pseudocyst was done for the first time in 1995 by Kim et al. [45] The pathophysiology of pseudocysts development is still unclear. Predisposal factor are: shunt infections with microaerophilic or anaerobic bacteria, low grade sepsis, mute clinical peritonitis, iterative shunt revisions, history of abdominal surgery, hyperproteinnorrachia, impaired absorption of the peritoneum, and silicone allergy. [46, 47]

In a series containing 12 patients with CSF pseudocysts, Gaskill et al. found acute shunt infections in 16%, and in 41.6% history of shunt infections. [46]  Rainov et al. found active shunt infection in 30% patients. [43] Peritoneal response to an infraclinical shunt infection is isolation of the distal catheter by fibrous tissue, forming pseudocysts. Pseudocysts suggest a self–limitating character of the infection. [46, 47]  The histopathology of the cystic ‘wall’ is fibrous tissue without epithelial lining, proving that the pseudocyst is secondary to a local inflammatory response. 

Pseudocyst standard recommended treatment, if there are no signs of infection, is removal of the distal catheter with or without resection of the cystic ‘walls’, followed by insertion of a new catheter into the abdominal cavity with another location or conversion of the shunt into a ventriculocardiac one. Gaskill et al. proved in his series of patients that it is not mandatory to remove the cystic ‘walls’, because pseudocysts can solve spontaneously, once the catheter is took off. [46] As an alternative, Deindl et al. report cases of aspiration of the pseudocyst contents, through the proximal end of the peritoneal catheter, followed by ventriculocardiac shunting. [48] However, this has the disadvantage that peritoneal cavity cannot be inspected, and other associated pathology cannot be treated. Infected pseudocysts are treated like abscesses or peritonitis, with initial external ventricular drainage, antibiotherapy and ventriculocardiac shunt. [46]

We performed in both cases evacuation of the cyst, resection of the cystic ‘walls’, debridement, lavage and drainage of the peritoneal cavity. The drain tube was placed into the Douglas pouch, chosen because of its declining position. We collected CSF from pseudocyst for laboratory examination and transformed the shunt into external ventricular drainage. CSF cultures were sterile in both cases. We have chosen to perform an external ventricular drainage and later a ventriculocardiac shunt instead of inserting a new peritoneal catheter because, in both cases, we found important changes into the peritoneal cavity, with adherences, bride and fibrous reshuffling between abdominal viscera, that in our previous experience, shown that these patients are prone for recurrent pseudocysts. And besides all this, we took into consideration the high risk of having an infected shunt, in spite of sterile CSF cultures. Prior converting the external shunt into ventriculocardiac one, patients received systemic antibiotherapy.

In our group CSF ascites was found in one patient. CSF ascites is a rare complication of VP shunts. [49] Pathogenic factors are: impaired absorption of the peritoneum, excessive production of CSF, hyperproteinnorrachia, shunt infections and tumoral seeding. 

CSF ascites may occur years after a VP shunting procedure. So far there are no sufficient explanations of this phenomenon. According to most authors, CSF ascites seems to be commonly related to tumors of the suprasellar region, optic pathway gliomas and craniopharyngiomas. [49, 50]  They lead to electrolytic abnormalities with hypernatremia and osmoreceptor dysfunctions, hyperproteinorrachia, and inappropriate secretion of antidiuretic hormone with plasma hypoosmolality. [51, 52]  But only hyperproteinorrachia is not enough to produce CSF ascites. [47]  Hyperproteinorrachia cannot cause ascites by its self unless protein level from ascites fluid is higher than protein plasmatic level. Infection was not proven to cause ascites, in spite of high cellularity of the CSF [53, 54], but shunt infection cannot be excluded from the ethiopathogenesis of ascites.

Chidambaram et al. performed a biopsy of the peritoneum in children with CSF ascites, and histological examination revealed granulation tissue infiltrated with numerous eosinophils, plasma cells, lymphocytes and histiocytes, focal areas of reactive mesothelial proliferation and vascular congestion. [49]

It is important to distinguish between CSF pseudocyst and ascites, two different entities with different pathogenesis, clinical presentations, and management strategies. [55] In pseudocysts infection pays an important role. The etiopathogenesis of pseudocysts probably is shunt infection with microaerophilic or anaerobic bacteria, low grade sepsis, or mute clinical peritonitis, which leads to epiploon and viscera agglutination around infected catheter. CSF ascites is most likely to be secondary to an impaired absorption of the peritoneum. 

Recommended treatment consists of distal catheter removal, and if no infection is found, a ventriculocardiac shunt placement. Ventriculopleural shunt is forbidden because, like peritoneum, pleura is also a mesothelial structure, and ventriculopleural shunting is followed by hydrothorax. If infection is present an external ventricular drainage is used, antibiotherapy, followed by ventriculocardiac shunting. If infection is found, aggressive antibiotherapy is mandatory. Yount et al. report ascites remission only with conservative treatment. [53] On the contrary, noninfected ascites require conversion into ventriculocardiac shunt. In our patient presenting with peritoneal irritation syndrome, we performed diagnostic laparoscopy that found ascites with clear, sterile CSF. We chose next to convert the system into a ventriculocardiac shunt. 

Immediate and aggressive treatment is required in patients with CSF ascites, because it can generate life– threatening complications, such as shock, sepsis, dyspnea or hepatorenal failure.

In our study group, one patient developed inguinal hernia without hydrocele. Inguinal hernia with or without associated hydrocele occurs in patients with ventriculoperitoneal shunts with a frequency of 3.8-16.8%. [3]  Clarnette et al. consider that patient's age pays an important role in occurrence of this particular abdominal complication. 30% of inguinal hernias occur in infants 0–3 month old, and 10% in children 1 year old. [3, 56]  Predisposal factors are: persistence of processus vaginalis (the patency of processus vaginalis can persist in 60–70% in infants during the first 3 months, in 50–60% in children 1 year old and up to 40% in children 2 years old), increased abdominal pressure, impaired absorption of the peritoneum, and tumoral seeding. [57, 58]

The treatment of choice is surgery. Hernioraphy can be performed laparoscopically with good results. Via laparoscopic approach bilateral exploration can be done, because in 50% cases hernias are bilateral. [59, 60]. Cases with associated early hydrocele may solve spontaneously within 1 month. Persistence of the hydrocele after 1 month requires surgery. We performed hernioraphy and exploration of the whole abdominal cavity. We found no contralateral hernia. VP shunting system was completely functional, so we decided to left it in place.

Shunt malfunction was caused in one patient by excessive length of the intraperitoneal tube. Shortening of the tube via laparoscopic approach was an easy and elegant treatment.

### Clinical findings

Shunt dysfunctions are followed by acute hydrocephalus. This is the reason why all patients presented signs of elevated intracranial pressure, headache, vomiting and loss of consciousness. Plus, 35.29% presented cranial nerve palsy, abducens palsy and upward gaze palsy, highly suggestive for acute hydrocephalus. The most common abdominal symptom was abdominal pain presented by 64.70% of the patients.

Although CSF passage through subcutaneous fibrous connective tissue sheath after shunt disconnection and migration was found by other authors, all of our patients developed active hydrocephalus. [61]

### Outcome

All abdominal complication can be solved laparoscopically, considering the 0% rate of conversions to open surgery. One patient, with medulloblastoma died, not of abdominal causes. Massive tumor recurrence was the main cause of patient's death. This last patient had shunt disconnection with distal catheter migration. We inspected peritoneal cavity, found and extracted the catheter. We place a new distal catheter into the peritoneal cavity. The patient had spinal and supratentorial secondary disseminations, but at autopsy we found no abdominal seeding along the catheter. From our former experience we consider this thing possible, but rare.

### Advantages of laparoscopy

Laparoscopic treatment decreases risks of an open surgery and diminishes adherence formation. Laparoscopy allows direct view the CSF flow through the distal catheter, catheter repositioning, peritoneal cavity exploration, viscerolisis, and treatment of associated lesions. Results following laparoscopic surgery are similar to those from open surgery. [41, 45]  Laparoscopy can also be used for distal catheter insertion or repositioning when we suspect important adherence syndrome.

### The effect of CSF over the peritoneum

Adherence syndrome, encountered in some patients, without any other abdominal pathology, in the absence of infection, make us believe that it is the result of low, prolonged local inflammatory response of the peritoneum to CSF. We consider prolonged CSF flow to be an irritating factor for the peritoneum.

## Conclusion

Abdominal complication following VP shunt can be successfully performed laparoscopically. Although the number of abdominal complication following VP shunt, which requires surgical treatment, is low, they raise problems regarding positive and differential diagnosis. In patients with abscesses, peritonitis, CSF ascites and selected cases of with pseudocysts, repositioning of the distal catheter is needed, frequently as a ventriculocardiac shunt. There are abdominal complications with no indication for surgery, like peritoneal irritation syndrome and CSF ascites. Free– disease interval varies from days (peritoneal irritation syndrome, abscesses) to month–years (pseudocyst, ascites), according to type of complication. Laparoscopic approach allows treatment and if shunt is fully functional to salve the existing shunt and avoid the potential morbidity associated with additional VP shunt placement. [2] Via laparoscopic approach the whole abdominal cavity can be inspected and any associated pathology treated.
